# Effect of COVID-19 on the Burden and Profile of Orthopaedic Patients Admitted Post-Resumption of Routine Services in a Tertiary Care Hospital in Delhi

**DOI:** 10.7759/cureus.44074

**Published:** 2023-08-24

**Authors:** Sunil Kumar, Anil K Jain, Khan A Maroof, Aditya N Aggarwal, Rajesh Arora, Ish K Dhammi, Himanshu Gupta

**Affiliations:** 1 Orthopaedics, University College of Medical Sciences, New Delhi, IND; 2 Community Medicine, University College of Medical Sciences, New Delhi, IND

**Keywords:** complication of treatment, missed, disease burden, orthopaedic, covid-19

## Abstract

Purpose

On resumption of routine services post-lockdown during coronavirus disease 2019 (COVID-19), we expected a backlog of orthopaedic patients who could not get appropriate and timely care and would now present with complications due to missed or delayed treatment. This study aimed to quantify the effect of COVID-19 on the burden and profile of orthopaedic patients admitted post-resumption of routine services.

Materials and methods

Data on all the patients admitted to the orthopaedic department were collected using an interviewer-administered schedule for a complete one-year period after the resumption of routine orthopaedic services in a tertiary care hospital in Delhi. For comparison of the burden of trauma patients with that during the pre-COVID-19 period, data were obtained from a similar study done on trauma patients in 2017 at the same institution. For patients with non-traumatic conditions, previous hospital records were used.

Results

A total of 1585 patients were admitted during the one-year period post-resumption of routine services following COVID-19 restrictions, which was 41% less than that compared to the corresponding pre-COVID-19 data. The proportion of patients from other neighbouring states showed a decline from 52% in the pre-COVID-19 period to 41.55% when healthcare services resumed during the COVID-19 period. Out of all admitted trauma patients in 2021, 12.7% presented with a missed or complication of treatment as compared to 3.1% in the pre-COVID period. Around half of them (52.5%) attributed their complications to a COVID-19-related lockdown.

Conclusion

There was a significant decline in the number of patients post-resumption of routine orthopaedic care services. Converting whole tertiary care teaching hospitals to COVID-19-dedicated hospitals must not be done as it leads to an increase in missed or complication of orthopaedic treatment.

## Introduction

Severe acute respiratory syndrome coronavirus 2 (SARS-CoV-2), a severe acute respiratory syndrome virus, first emerged in Wuhan, China, in December 2019 [1]. The World Health Organization (WHO) then proclaimed this coronavirus to be a global health emergency, calling for international cooperation and a concerted effort to combat this pandemic as the viral outbreak expanded to nations on several continents [2]. The pandemic caused large-scale mortality and morbidity and disrupted lives on a global scale. India reported its first case on January 30, 2020, which was followed by a sharp increase in the number of cases [3]. The healthcare delivery system was overwhelmed due to the sudden surge of COVID-19 cases. Patients suffering from diseases other than coronavirus disease 2019 (COVID-19), such as orthopaedic patients, faced a delay in treatment due to the shift of healthcare facility priorities towards COVID-19 patient management. Since more beds in wards and intensive care units had been assigned to COVID-19 patients, many surgical departments reported a decline in the number of surgeries conducted [4].

The imposition of lockdown as a strategy to flatten the curve of COVID-19 patients also delayed the management of these patients. More so, converting certain major public teaching hospitals in Delhi providing tertiary care to COVID-19-dedicated hospitals, including the hospital where this study was conducted, appeared to have the highest impact on the timely management of orthopaedic patients. This inevitably disrupted the orthopaedic care and treatment pathways and protocols at both elective and emergency services, affecting access and availability of treatment services [5]. OPD attendance went to zero, and elective surgeries were cancelled during this period. Similar scenarios were also witnessed in other countries as well, such as in Italy, where OPD and ER consultations decreased by almost 3/4th and elective OTs were shut down completely as many orthopaedic surgeons were redirected to the care of COVID-19-infected patients [6].

On the gradual resumption of routine orthopaedic services in our teaching hospital after the first wave, there was a decline in COVID-19 cases and relaxation in lockdown. It was expected that there would be a backlog of patients who could not get appropriate and timely orthopaedic care. Estimates were calculated for the backlog in the US, which stated that in the optimistic scenario, there will be a cumulative backlog of >1 million surgical cases two years after the end of elective surgery deferment [7].

Deferring care to orthopaedic patients during lockdown led to high orthopaedic disease burden, morbidity, and disease-related sequelae, which will present in the hospital once services resume [8].

For the initial wave of the pandemic, health systems were not completely prepared, and different nations implemented social interventions at various times and to varying degrees. Therefore, researching how the pandemic affected the practice of orthopaedic surgery in various nations will aid health systems in better preparing for pandemics in the future, protecting public health, and enabling hospitals to continue offering top-notch orthopaedic care [9].

We therefore aimed to quantify the effect of COVID-19-related lockdown and the conversion of public teaching hospitals to COVID-19-dedicated hospitals on orthopaedic admissions.

## Materials and methods

This cross-sectional study was conducted in the Department of Orthopaedics of a public health tertiary care hospital in the national capital region of India from January 2021 to April 2022 after institutional ethics committee human research clearance (IECHR/2020/PG/47/37)

Post-resumption of routine services after the first wave of COVID-19 and when the lockdown was eased, all the patients who were admitted in the orthopaedics ward for a period of one year, i.e., from January 21st, 2021, to April 21st, 2022 (excluding the months of April, May, and June of 2021, in which the hospital was again dedicated to COVID related patients in the second COVID-19 wave) and who were consenting to be a part of the study were included.

Information from these patients was obtained using an interviewer-administered semi-structured schedule, and patients were categorised into those who: i) had sustained the musculoskeletal injury less than 21 days before reporting to the hospital under study, ii) had sustained the musculoskeletal injury for more than 21 days before reporting to the hospital under study, and i) were admitted for non-traumatic orthopaedic conditions. Information was also obtained regarding their socio-demographic profile and their injury.

For comparison of the burden of trauma patients with that in the pre-COVID-19 period, data were taken from a similar study done in 2017 on the burden and profile of trauma and trauma-related patients in the same department and same institution in which the current study was carried out [10]. For data on patients with non-traumatic conditions, records of pre-COVID-19 duration from wards and operation theatres from the same institution in which the current study was carried out were obtained for comparison. Certain open-ended questions to find out the impact of lockdown and the cessation of routine orthopaedic treatment in the hospital were also part of the schedule.

The comparison of the burden and profile of admitted orthopaedic patients during the COVID-19 period with that of the pre-COVID-19 period could be done for nine months instead of the planned twelve months because for three months, i.e., from April to June 2021, the hospital was fully converted to a COVID-19 dedicated hospital, and routine orthopaedic services were stalled.

IBM SPSS Statistics for Windows, Version 20 (Released 2011; IBM Corp., Armonk, New York, United States) was used for statistical analysis. The total number of patients admitted in the study period is presented as counts. The categorical variables, such as the status of delay or the presence of complications among patients, are presented as proportions. The comparison of these variables in the duration after resumption of routine orthopaedic services during COVID-19 with that of pre-COVID-19 data was done using the chi-square test.

The response to an open-ended question regarding the effect of lockdown and cessation of routine orthopaedic care in hospitals was captured verbatim in the local language and translated to English. Rapid content analysis was done to identify the themes emerging from the data. Their findings are presented as themes and selected quotations.

## Results

A total of 1585 patients were admitted to the orthopaedic department over the period of one year. Out of these, 90.9% (1441/1585) were related to trauma, and 9.1% (144/1585) were related to non-traumatic aetiology. Out of 1441 patients, the majority (86.5%) presented within 21 days of the trauma, and 13.5% presented later than 21 days after the trauma.

Patients' ages ranged from 1.5 to 92 years, with a median age of 30 years. The majority of the patients, i.e., 75.6% (1199/1585), were males. The majority, 58.4% (927/1585), were from Delhi, 39.6% (629/1585) were from the nearest neighbouring state of Uttar Pradesh, and 1.8% (29/1585) were from other neighbouring states. 64.8% (1028/1585) of the patients in the study population were either unemployed or employed in unskilled or semi-skilled occupations. Around three-fourths (78.6%) were either illiterate or educated below the intermediate level.

Around two-fifths of patients, i.e., 19.4% (307/1585), presented with a missed or complication of treatment. Out of these, 57% (175/307) patients were those who reported trauma after 21 days, and 43% (132/307) patients were related to non-traumatic orthopaedic conditions. The majority, i.e., 61.24% (188/307) of these patients, did not receive any pre-hospital care, with 79.4% (139/307), 12.6% (12/307), and 8% (14/307) brought from home, accident site, and hospital, respectively. Around half, i.e., 52.5% (161/307) of these patients stated that they had difficulty seeking treatment due to the imposition of the COVID-19 lockdown. Most of these patients, i.e., 69.8% (214/307) were males, and most, i.e., 28.85% (89/307), had one or more known co-morbidities. The median age of these patients was 33 years.

The difference in the profile of orthopaedic patients admitted during the COVID-19 period and the pre-COVID-19 period is given in Table 1.

**Table 1:** Burden and profile of orthopaedic patients admitted during the pre-COVID-19 period (2017) and the COVID-19 period (2021) *Significant at P<0.05

	Pre-COVID-19 period	COVID-19 period	OR (95% CI)
Total number of patients	2029	1188	-
Patients from outside Delhi	1151 (56.7%)	494 (41.6%)	0.54 (0.47-0.63)*
Patients with trauma	1867 (92.0%)	1078 (90.7%)	0.85 (0.66-1.10)
Trauma patients presenting later than 21 days of trauma	305 (16.3%)	128 (11.9%)	0.69 (0.55-0.86)*
Trauma patients presenting with missed or complication of treatment	57 (3.1%)	122 (12.7%)	4.05 (2.93-5.60)*

On comparison with pre-COVID-19 period data, there was a reduction of 41.4% in the number of orthopaedic admissions during the COVID-19 period (n = 1188) as compared to the pre-COVID-19 period (n = 2029). We found a significant difference in the frequency of missed or complication of treatment among hospitalised trauma patients when comparing the pre-COVID and COVID periods. As opposed to 3.1% in the pre-COVID era, 12.7% of patients presented with such issues during the post-COVID period. An odds ratio (OR) greater than 1 was obtained.

**Figure 1:**
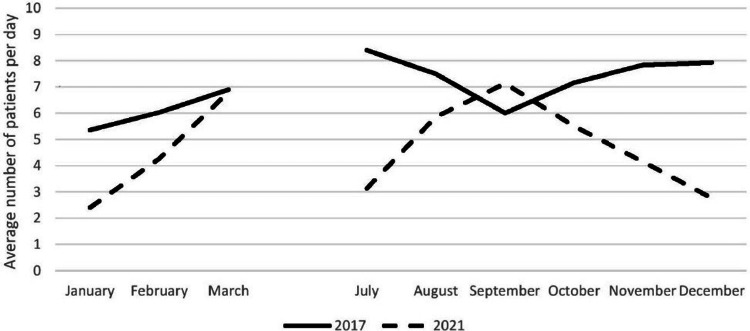
Month-wise daily average number of patients admitted in the orthopaedics department of GTB hospital over a period of one year (pre-COVID-19 period in 2017 and COVID-19 period in 2021) *The break in the lines corresponds to April to June 2021, when the hospital was converted to a COVID-19-dedicated hospital, and so comparisons for those months could not be done.

The comparison of the monthly average of orthopaedic admissions during the COVID-19 period with that of the pre-COVID-19 period is given in Figure 1.

The perceptions of orthopaedic patients regarding the effect of COVID-19 lockdown on their treatment-seeking behaviour are presented below as themes and some selected quotations.

A. Inappropriate treatment

"My wound got infected due to a lack of appropriate dressing at the local practitioner."

B. Delay in receiving treatment

"Due to the lack and shutdown of outpatient department services, I was not able to get the appropriate treatment at the time."

"I was denied admission due to the shutdown of the operation theatre (surgical services), due to which the severity of my disease has increased."

C. Development of complications

"My bone got united in a deformed position due to a lack of appropriate care."

D. Had to consult an osteopath or a local practitioner.

"I was treated by an osteopath due to the lack of expert doctors in my area."

"I was treated by a local physiotherapist near my home, and my pain got worse."

## Discussion

We found that the total number of patients admitted over the period of one year was 1585. This number is considerably lower as compared to pre-COVID-19 times in a period of one year. In a similar study done under similar circumstances on the burden of trauma-related patients at an Indian tertiary care centre, 2473 patients were included during the study duration of one year [10]. This was probably due to the phase-wise resumption of services, which was interrupted by the second and third waves of COVID-19, and may also be due to fewer motor vehicle accidents during the lockdown period. The reduction in trauma cases during COVID-19 was also evidenced by other studies [11]. Testa's study also found that high-energy trauma was reduced due to a reduction in high-energy traumatic occasions, but the number of home accidents involving the elderly remained static [11]. 

In the current study, the majority (91%) of patients were admitted due to trauma, and only 9% due to non-traumatic aetiology. This was because of the limited and phase-wise resumption of services. Non-trauma patients were admitted date-wise when scheduled for surgery, unlike trauma patients. In a study done in another tertiary care centre in a different region of the same country, it was found that there was a reduction of 62.6% in the number of orthopaedic-related trauma cases during the lockdown period [12]. According to a study done in Hong Kong, there was a 53% reduction in elective joint replacement procedures from January to June 2020 [13]. The number of orthopaedic patients waiting for surgery has dramatically increased as a result of elective surgery cancellations and postponements. Once the situation improves, hospitals should put specific plans in place to manage upcoming elective surgeries quickly and effectively [14].

There were patients belonging to all age groups, but the majority belonged to the young age group, signifying the involvement of the young population in dangerous work and road traffic accidents post-pandemic as well. This finding is supported by similar findings in studies done in the same institution before COVID-19 [10] and also in a study done by Bhat et al. [15]. The majority of the admitted patients were from the same state, i.e., Delhi. In pre-COVID-19 times, the flow of patients was higher from other states, as evidenced by a study done in 2017, in which it was found that the majority of patients admitted were from neighbouring states [10]. This might be due to decreased interstate movement and more stringent COVID-19 protocols in the state of Delhi, due to which patients were forced to visit hospitals in their home state only.

One-fifth of patients presented with missed or complication of treatment, the majority of them belonging to trauma patients presenting after 21 days of injury or non-trauma patients. These were the patients who missed their treatment or complicated their condition due to the unavailability of proper care due to the COVID-19 pandemic. These numbers were higher in the initial few days of resumption of services and declined afterwards but kept on happening as the hospital was shut down in between due to COVID-19 waves. In a similar study done on trauma patients in an Indian tertiary care centre, 13.8% of patients had a complication of treatment received prior [10]. In a study done by Tartarilla et al., telemedicine was found to be helpful in reducing missed care [16]. When comparing the pre-COVID and COVID periods, we discovered a statistically significant difference in the frequency of patients with missed or complication of treatment. In the post-COVID era, 12.7% of patients had such problems compared to 3.1% in the pre-COVID era, demonstrating an increased risk of complications during the COVID period.

Almost half (52.5%) of the patients with missed or complication of treatment were related to the lockdown imposed on the hospital by making it a COVID-19 dedicated facility and the lockdown that prevented movement of people from one place to another. In the open-ended questionnaire, the maximum number of patients mentioned lockdown as the main reason that led them to miss care or get treatment from unqualified personnel, due to which they landed up in complicated situations like malunion and nonunion. This was also evidenced in a study done on the effect of lockdown on chronic diseases, in which it was found that patients missed regular testing and routine health checkups due to COVID-19 [17]. In our study, we found that a lesser number of patients came from neighbouring states during the COVID-19 period as compared to the pre-COVID-19 period. This was expected, as during COVID-19 there were travel and other restrictions to contain the spread of the pandemic in the community.

Limitations of the study

This study includes only those orthopaedic patients who reached the hospital for admission. This study was conducted in a public teaching hospital primarily serving people of lower socioeconomic status in the community. The comparison with the pre-COVID data could only be done for limited months as routine services were curtailed during the second COVID wave for three months, i.e., April to June 2021.

## Conclusions

COVID-19 related disruption led to a huge decline in the number of orthopaedic patients admitted to the hospital even after the resumption of routine services during the pandemic period. Patients coming from neighbouring states witnessed a greater decline, and more patients presented with missed or complication of treatment. Patients perceived that their orthopaedic conditions worsened due to disruption of routine health services, possibly due to COVID-19 lockdown and converting the whole hospital to a COVID-19-dedicated one.
